# Nature-Guided Synthesis of Advanced Bio-Lubricants

**DOI:** 10.1038/s41598-019-48165-6

**Published:** 2019-08-12

**Authors:** Trevor Romsdahl, Asghar Shirani, Robert E. Minto, Chunyu Zhang, Edgar B. Cahoon, Kent D. Chapman, Diana Berman

**Affiliations:** 1 BioDiscovery Institute and Department of Biological Sciences, University of North Texas, Denton, TX USA; 2 Department of Materials Science and Engineering, University of North Texas, Denton, TX USA; 3 Department of Chemistry and Chemical Biology, Indiana University-Purdue University Indianapolis, Indianapolis, IN USA; 4 National Key Lab of Crop Genetic Improvement and College of Plant Science and Technology, Huazhong Agricultural University, Wuhan, China; 5 Center for Plant Science Innovation & Department of Biochemistry, University of Nebraska-Lincoln, Lincoln, NE USA

**Keywords:** Natural products, Plant sciences

## Abstract

Design of environmentally friendly lubricants derived from renewable resources is highly desirable for many practical applications. Here, *Orychophragmus violaceus* (Ov) seed oil is found to have superior lubrication properties, and this is based on the unusual structural features of the major lipid species—triacylglycerol (TAG) estolides. Ov TAG estolides contain two non-hydroxylated, glycerol-bound fatty acids (FAs) and one dihydroxylated FA with an estolide branch. Estolide branch chains vary in composition and length, leading to their thermal stability and functional properties. Using this concept, nature-guided estolides of castor oil were synthesized. As predicted, they showed improved lubrication properties similar to Ov seed oil. Our results demonstrate a structure-based design of novel lubricants inspired by natural materials.

## Introduction

Increasing transportation and other industrial activities since the beginning of the last century have consumed much of the world’s non-renewable petroleum-based energy resources, and a significant portion of the energy produced is spent in overcoming the friction of moving mechanical systems^1,2^. Many research efforts are dedicated to the understanding of the fundamental mechanisms of friction, and creating new ways to achieve higher efficiency and longer durability in all types of sliding, rolling, or rotating contacts^3,4^. Introducing an oil lubricant into the sliding contact is the commonly-used and most effective method for reducing friction and wear, prolonging the lifetime of today’s moving mechanical assemblies^5^. Lubricants reduce friction by reducing sliding contact interfaces from metal-to-metal contacts or by forming a low-shear, high-durability boundary film on rubbing surfaces^6^. The petroleum industry offers a wide range of lubricant compositions exhibiting targeted physical and chemical characteristics for specific applications.

Use of conventional and synthetic oils and their products is often associated with producing hazardous waste and dangerous exhaust^7^. While being effective for lubrication applications, synthetic oils and their derivatives often are not appropriate for a range of bio-friendly applications, such as those in marine, food and medical industries; in addition, synthetic oils lead to adverse impacts to the environment^8^. Petroleum-based oils usually exhibit a low flash point leading to instability of lubrication properties and rapid degradation during thermal cycling^9^.

In an effort to design better lubricants that are environmentally-friendly, nature has provided inspiration. Plant-based oils often naturally demonstrate excellent lubrication characteristics, whereas lubrication with conventional and synthetic oils requires blending of several selected base oils with additive(s). Rapeseed-based lubricants are widely used in food and detergent manufacturing^10,11^. Jojoba oil has been tested as a blending component in lubricating oil formulations to improve their viscosity, anticorrosion and antifoaming properties^12,13^. Castor is one of the oldest cultivated crops for vegetable oil production, and a source of a hydroxy fatty acids (hFAs), which makes its production extremely important to the global chemical industry^14,15^. Compared to standard lubricants, castor oil demonstrates higher viscosity, density, thermal conductivity, and pour point values. Castor oil has also been suggested as a base oil for making 100% biodegradable greases and oleogels^16^. However, the origin of functional characteristics of the bio-oils is poorly understood and their use is limited to as-received, cold-pressed or refined liquids. Improvement in the lubrication characteristics of renewable lubricants is, therefore, highly desirable with multiple efforts being dedicated to this goal^17–19^.

Recently, dihydroxy FAs were described in an obscure Brassicaceae species, *Orychophragmus violaceus*, as nebraskanic acid (7,18-(OH)_2_-24:1^Δ15^) and wuhanic acid (7,18-(OH)_2_-24:2^Δ15,21^)^20^. Preliminary studies suggested this oil possessed excellent lubricity properties at high temperature, likely due to the unusual dihydroxy fatty acid content of the oil. However, we show here that it is not the hFA content *per se*, but rather the presence of oligomeric estolides naturally found in the seed oil that affords its superior lubricity properties.

The OH functional groups of hFAs can be exploited for industrial applications such as by esterifying other FAs to the free OH groups to create estolides or by polymerization of FAs through ester linkages between hFAs. Synthetic estolides can be produced chemically by acid-catalyzed reactions or by reverse catalysis with lipases to drive the esterification of hFAs^21,22^. Varying the type of FAs in estolide synthesis changes the properties of the resulting estolide-containing oil that may improve or alter the properties for a desired application. The value of estolides comes from their higher oxidative stability, greater lubricity, and low temperature properties compared with typical petroleum-based oils^23^. Previously, estolides of various types have been found to occur naturally in plants that produce hFAs including ricinoleoyl estolides from castor, estolide TAGs in various *Physaria* species, *Heliophila amplexicaulis*, *Mallotus philippensis*, *Trewia nudiflora*, *Chamaepeuce afra*, and *Sapium sebiferum*, and an estolide of digalactosyldiacylglycerol from *Avena sativa*^24–29^. However, the amounts of estolides in these examples are generally quite low, and rarely do polymeric TAG estolides accumulate in seed storage tissues. By contrast, in this study, naturally occurring TAG estolides were found to comprise the entirety of the seed oil content from *Orychophragmus violaceus* (Ov). Additional detailed tribology analyses of Ov oil and fractions of capped and uncapped estolides from the seed oil indicated greater thermal stability, oxidative stability, and lubricity compared to castor oil at a wide range of temperatures. Further, the structure-function relationship in naturally occurring TAG estolides of Ov guided the improvement of bio-based lubricants for environmentally acceptable industrial applications.

## Results and Discussion

**Figure 1:**
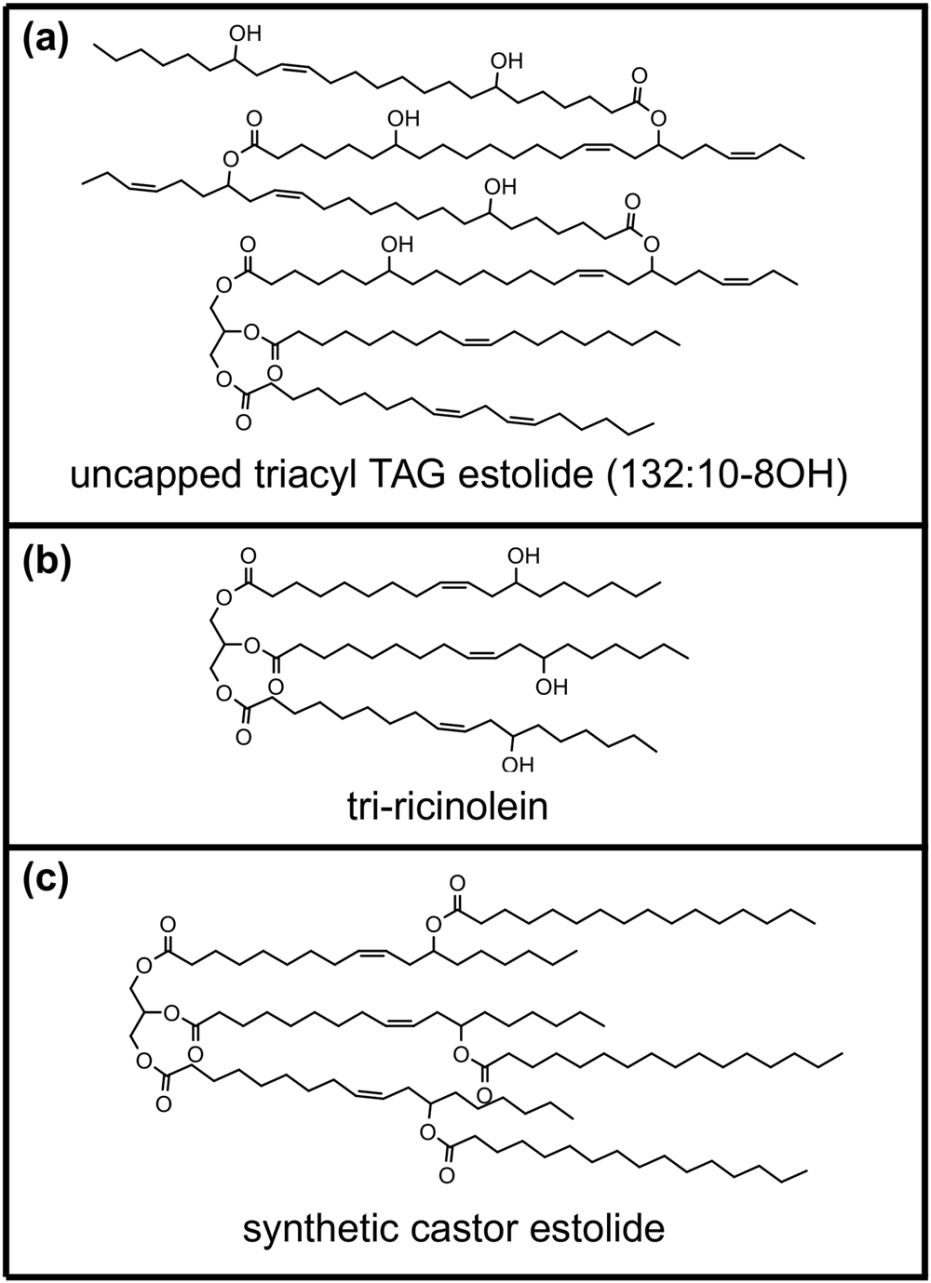
Biobased lipids of interest. (**a**) A representative TAG estolide from Ov seed oil consisting of a triacyl TAG estolide (132:10-8OH, same as in Fig. 5b). (**b**) Tri-ricinolein, the major hydroxy TAG of castor oil with three ricinoleate moieties. (**c**) A representative of a synthetic estolide made from castor oil with the base hydroxy TAG tri-ricinolein and palmitoyl moieties added to each OH (same as in Fig. 7e; also compare 1b and 1c).

**Figure 2:**
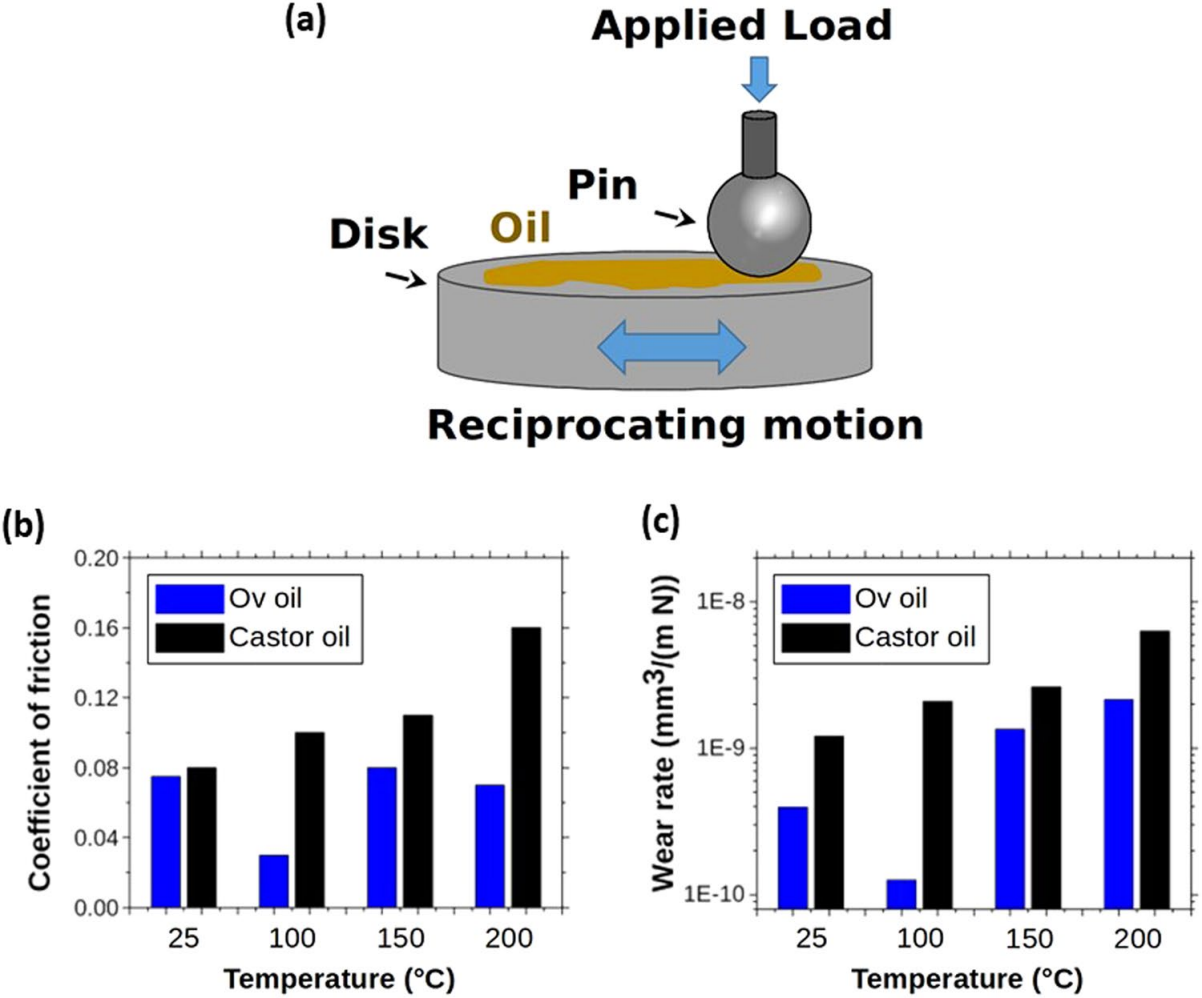
Lubrication characteristics of Ov oil and castor oil. (**a**) Schematic of experimental setup. Coefficient of friction (CoF) for the tribological tests performed with Ov oil and castor oil at different temperatures for the duration of 3000 cycles. (**b**) CoF measurements for Ov and castor oils at 25 °C, 100 °C, 150 °C, and 200 °C. (**c**) Wear rate of steel surfaces used in CoF measurements for both Ov and castor oils.

Oil pressed from Ov seeds showed superior performance characteristics compared with the industry standard castor oil. For comparative purposes, the structures of *O. violaceus* TAG estolides, castor TAGs, and synthetic castor TAG capped estolides are shown in Fig. 1. Both oils demonstrated good wetting characteristics of the steel surfaces (Supplemental Fig. 1). The friction and wear of steel surfaces lubricated with Ov oil in comparison to castor oil showed lower values at all temperatures measured (Fig. 2, Supplemental Figs 2, 3). The coefficient of friction (CoF) for Ov oil showed up to a threefold reduction at elevated temperatures. For all temperatures, the wear rate of Ov oil lubricated steel surfaces was less by at least an order of magnitude (Fig. 2).

**Figure 3:**
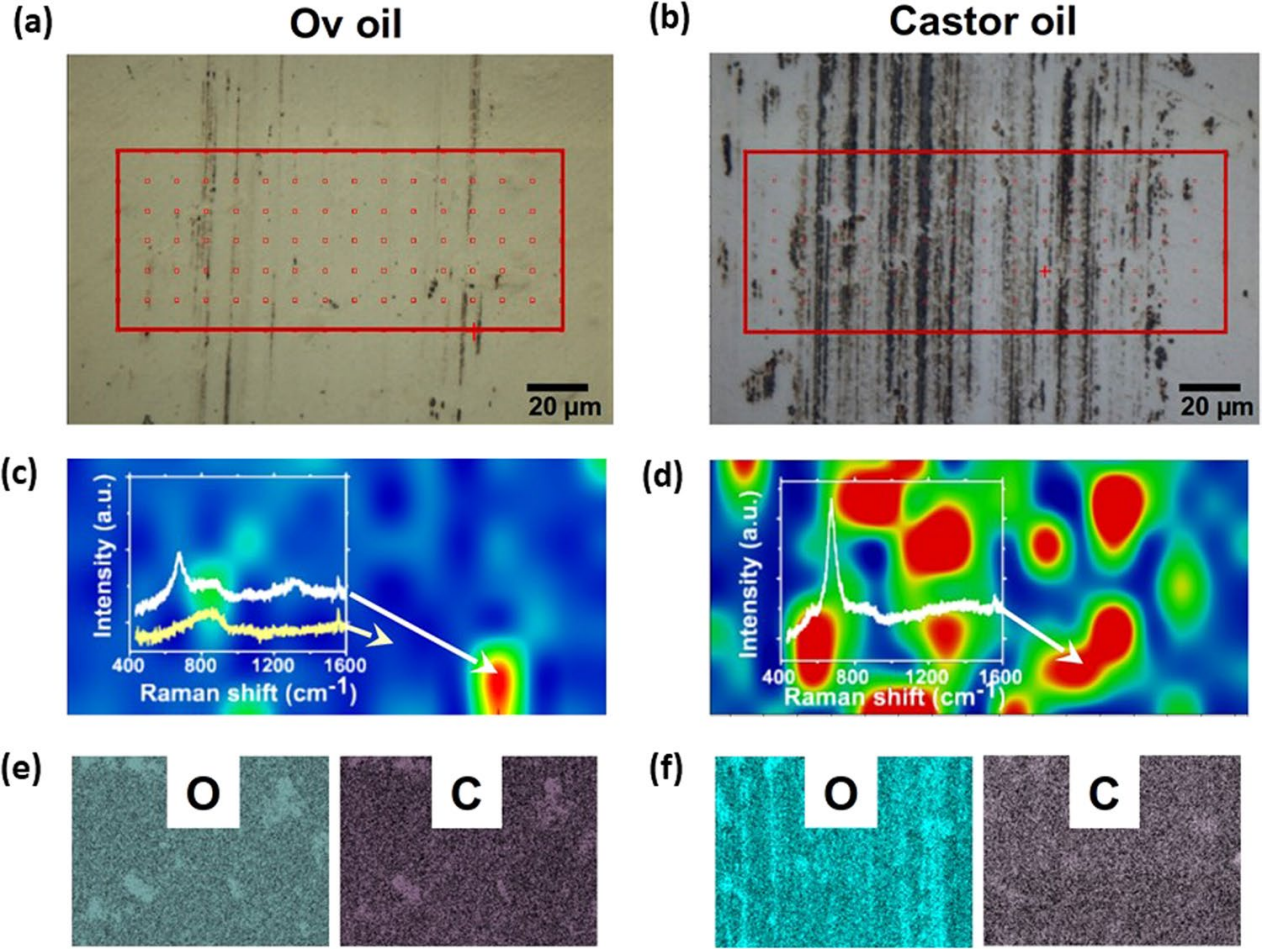
Analysis of the wear tracks formed in Ov and castor oil. Analysis of the wear track formed after the 100 °C tribotest of sliding steel surfaces lubricated with Ov oil (**a**) and castor oil (**b**). Raman 2D map of the iron oxide peak (at ~675 cm^−1^) of Ov oil (**c**) indicates very little oxidation of the steel surface during sliding in contrast to castor oil (**d**). Detailed EDS analysis of oxygen, O, and carbon, C, for the wear tracks formed during lubrication with Ov oil (**e**) and castor oil (**f**) further confirms the better oxidation resistance and protection properties of Ov oil.

To better understand the protective properties of Ov oil, the wear tracks formed with Ov oil at 100 °C were compared to those generated with castor oil (Fig. 3). Raman mapping of the iron oxide peak at ~675 cm^−1^ indicated much higher corrosion of steel inside the wear track formed during sliding in castor oil (Fig. 3d). In contrast, Ov oil demonstrated excellent protection against oxidation of the surface, which was attributed to the degradation resistance of the oil and uniformity of the protective lubricative layer (Fig. 3c). Scanning electron microscopy energy dispersive x-ray spectroscopy (SEM-EDS) analysis of the wear track confirmed the oxidative resistance of metals lubricated with Ov oil (Fig. 3e,f, Supplemental Fig. 4). Together, these results suggest that Ov oil enables suppression of steel surface oxidation under high contact pressure and shear conditions and leads to better lubricative characteristics of the oil. Interestingly, no carbon contrast is observed inside the wear track, indicating that Ov oil lubricity originates from the oil itself rather than from tribochemically driven formation of protective layers^30–32^. These results suggest that Ov oil shows high stability to variation in the local heating induced by the applied stresses a the sliding interface.

**Figure 4:**
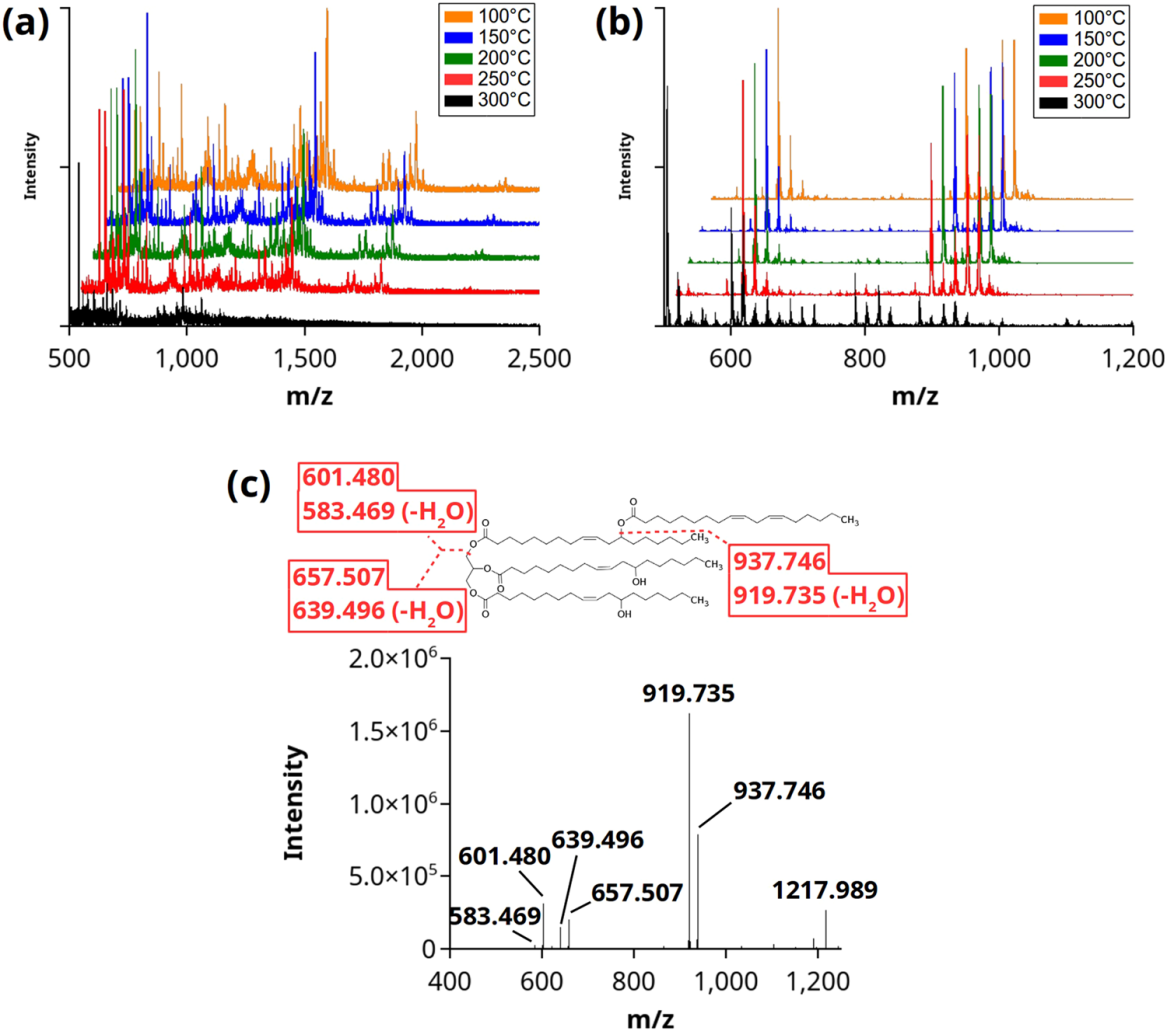
Thermal stability of Ov and castor oils. ESI-MS analysis of Ov (**a**) and castor (**b**) oil from 100 °C to 300 °C. At 300 °C, castor oil appears to show fragmentation. High-mass *m/z* peaks appeared in castor oil at 300 °C, such as at *m/z* 1217.991, further analyzed with MALDI-MS/MS (**c**) and found to be an oleoyl estolide of tri-ricinolein.

To further assess thermal stability of Ov and castor seed oils, electrospray ionization mass spectrometry (ESI-MS) was used to determine how the components of each oil changed following exposure to temperatures ranging from 100 °C to 300 °C (Fig. 4). Few changes were seen in either Ov or castor oils from temperatures 100 °C to 250 °C. At 300 °C, most of the *m/z* peaks from Ov seed oil were reduced or absent (Fig. 4a). In contrast, castor oil showed a marked decrease in hydroxy TAG content at 300 °C, from *m/z* 870 to 960 (Fig. 4b). Diacylglycerol *m/z* peaks appeared to increase in intensity from *m/z* 610 to 730. These observations suggest thermal degradation of castor oil occurs by fragmentation of glycerol-bound FAs. Several other peaks increased in intensity just below the *m/z* range of hydroxy TAG from 780 to 840. Additionally, peaks between *m/z* of 1050 and 1250 increased. The *m/z* peak at 1217.991 was selected for matrix assisted laser desorption ionization (MALDI)-MS/MS analysis to determine the identity of this novel peak present only at 300 °C treated oil but absent at lower temperatures. Fragmentation of the parent ion suggested a TAG estolide of tri-ricinolein with an additional linoleate esterified to one of the hFAs (Fig. 4c).

**Figure 5:**
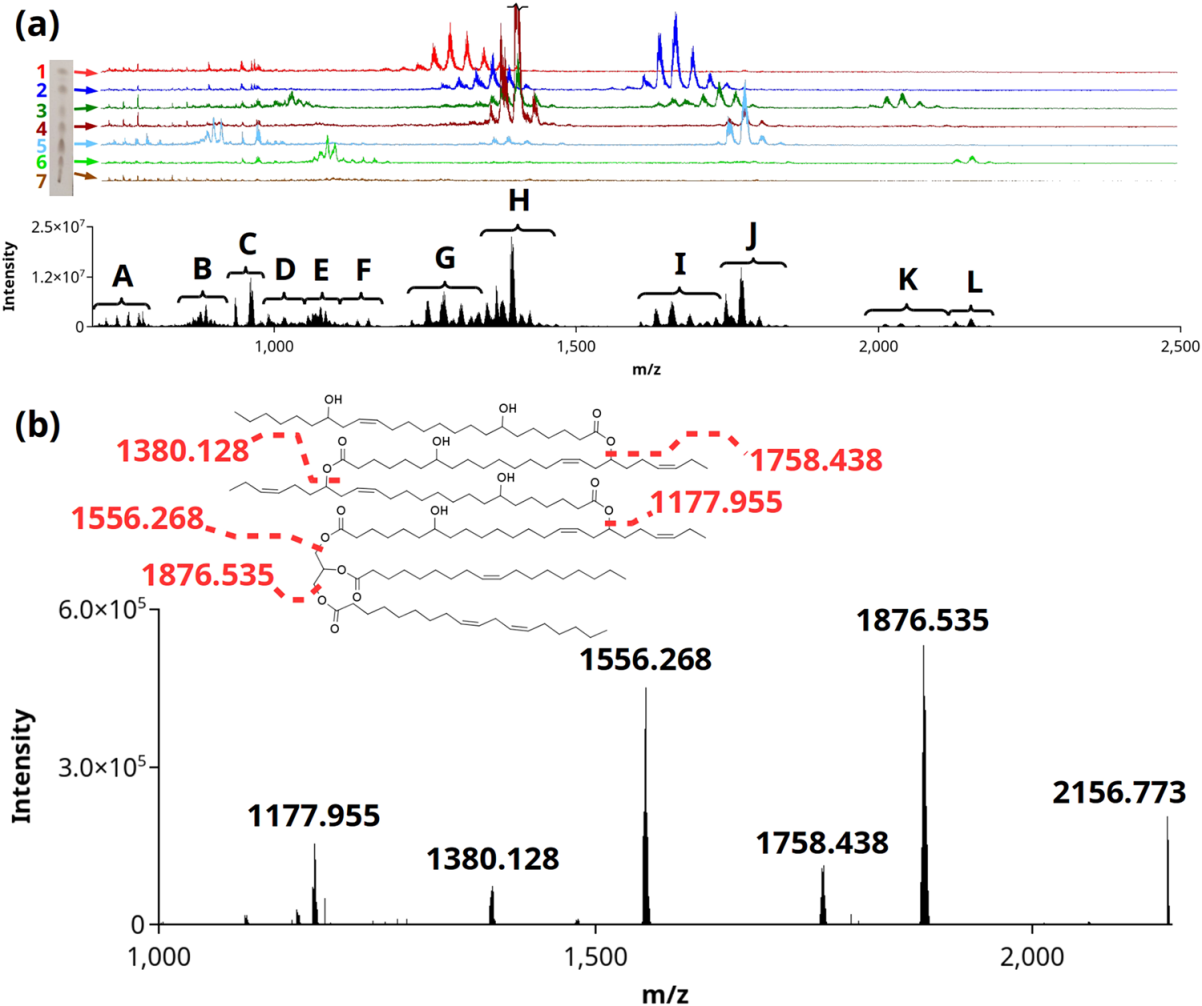
Ov TAG estolide structure characterization. HPTLC (a, left) separated Ov oil into six bands: (1) capped monoacyl TAG estolide (red), (2) capped diacyl TAG estolide (dark blue), (3) capped triacyl TAG estolide (dark green), (4) uncapped monoacyl TAG estolide (dark red), (5) uncapped diacyl TAG estolide (light blue), and (6) uncapped triacyl TAG estolide (green). ESI-MS analysis of Ov seed oil showed peaks of high *m/z* values (**a**, black and below). The labels G-L denote to ions corresponding to the main species in each isolated HPTLC band (mass spectral traces are plotted next to the HPTLC in the stated color). Further MS/MS analysis and *m/z* values showed that the remaining species were present during the analysis of the crude oil: (A) PC and diacyl estolide fragments, (B) doubly charged diacyl TAG estolide, (C) hydroxy TAG as possible in-source fragmentation, (D) doubly charged capped triacyl TAG estolide, (E) doubly charged uncapped triacyl TAG estolides, and (F) triacyl estolide fragments. (**b**) MALDI-MS/MS of the uncapped triacyl TAG estolide 132:10-8OH shows fragmentation at glycerol and estolide ester linkages. Full-length HPTLC image is presented in Supplemental Fig. 11.

The superior lubrication properties of Ov oil likely were derived from chemical structures of its components, and, while the oil was known to contain dihydroxy fatty acids, analysis of the oil by ESI-MS revealed a complex mixture of TAG estolides with many high-mass *m/z* values not generally encountered in other seed oils (Fig. 5). These *m/z* values were far higher than the calculated values of TAG or hydroxy TAG containing nebraskanic or wuhanic acid moieties. High-performance thin layer-chromatography (HPTLC)-separated Ov oil revealed that essentially all of the oil was in the form of TAG estolides of high *m/z* (Fig. 5a). ESI-MS/MS and MALDI-MS/MS confirmed the presence of TAG estolides, and a representative analysis by high-resolution MS on a MALDI-LTQ orbitrap mass spectrometer showed one of the abundant species at *m/z* 2156.773 as having three dihydroxy very long chain fatty acids (VLCFAs) esterified to a glycerol-bound dihydroxy VLCFA (Fig. 5b). The parent ion mass of *m/z* 2156.8 indicated a sodiated uncapped triacyl TAG estolide of 132:10-8OH (132 FA carbon atoms: 10 total double bonds: and 8 total hydroxy substitutions). Strong fragment ions were found at *m/z* 1876.535, 1758.438, 1556.268, 1478.202, 1380.128, and 1177.955. Neighboring fragment ion peaks differing by approximately 2 amu suggested overlapping isobaric molecular species within the MS/MS spectrum (Fig. 5b). The *m/z* fragment peaks at 1177.955 and 1556.268 both suggest a single glycerol-bound hFA with an estolide branch chain rather than multiple hFAs bound to the glycerol backbone. The most abundant diacyl TAG estolide showed a similar arrangement with normal FAs on two of the carbons in the glycerol backbone and a branched estolide on a dihydroxy VLCFA esterified to the third carbon on the glycerol backbone (Supplemental Fig. 5).

**Figure 6:**
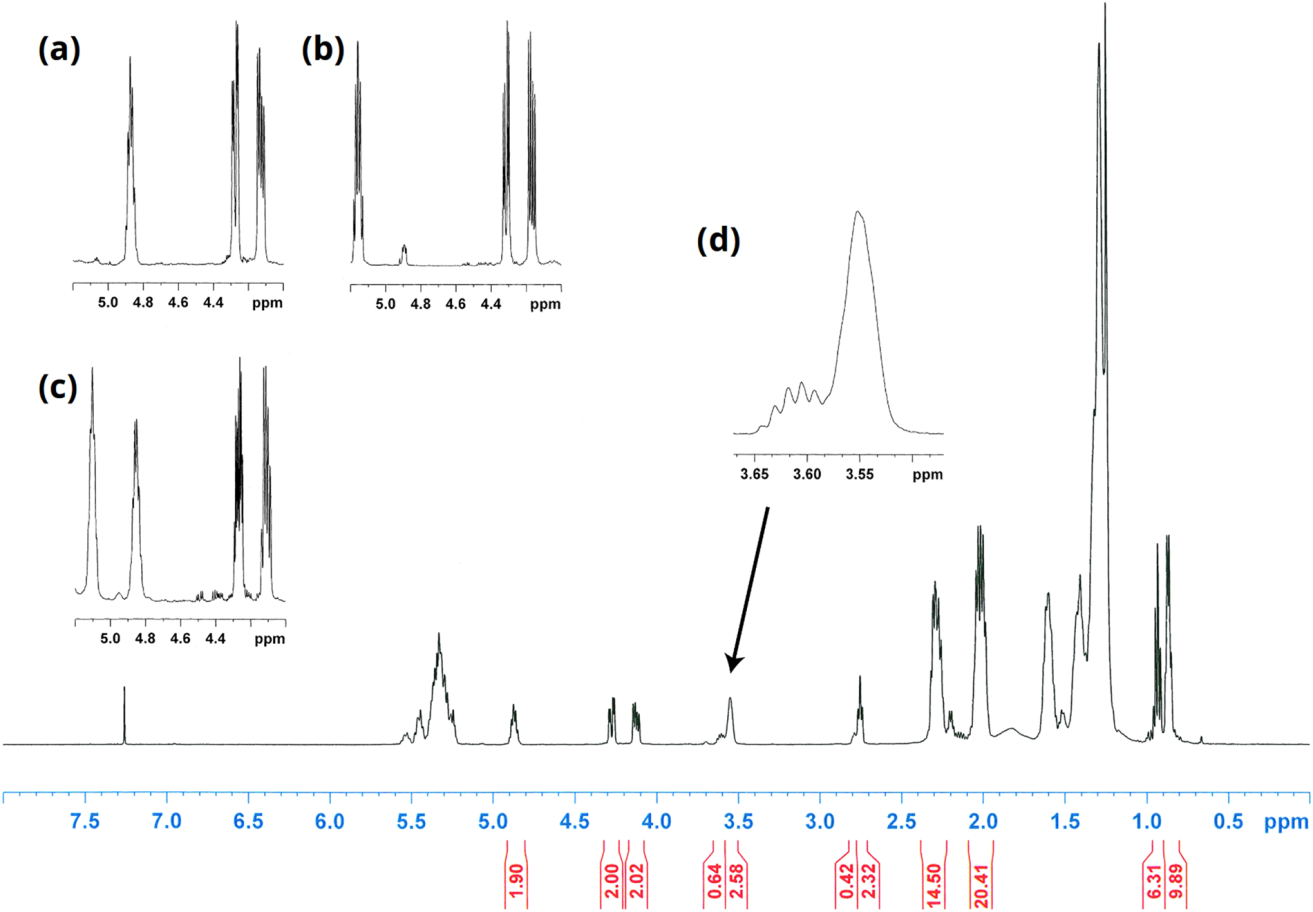
Complete 500-MHz proton NMR spectrum for unfractionated Ov estolide oil in CDCl_3_ at 28 °C. Full spectrum (bottom) shows estolide, glycerol methylene, and hydroxy-bearing methine resonances within the region from δ 5.24 – 3.5 ppm, as well as other signals corresponding to the acyl chains. (**a**–**d**) Show an expansion of the methine on the carboxyl side of an estolide ester (δ~4.9) and the doublet of doublet (dd) resonances for the methylene hydrogens of the glycerol backbone at approximately δ 4.1 and 4.3 ppm. (**a**) Expansion for Ov oil with the estolide signal at δ 4.86. Spectral data is shown for benzoylated castor oil (**b**), where the homoallylic estolide methine at C-12 is further downfield at δ 5.15 but the glycerol resonances are unperturbed. In benzoylated Ov oil (**c**), both naturally occurring and benzoyl estolides linkages are observed. Of note, signals from the glycerol backbone are more complex, altered by the proximate benzoyl ester at C-7, possibly stemming from magnetic anisotropy. In panel (d), the smaller, downfield multiplet for the methine of the free C-18 alcohol and the prominent broad methine resonance at C-7 for the free hydroxyl group in Ov oil are observed, consistent with the estolides primarily occurring at C-18 on wuhanic/nebraskanic chains.

Proton NMR data for the unfractionated estolide oil displayed considerable similarity to the spectra for methyl wuhanate, the methyl ester derived from the most abundant acyl chain in Ov oil^20^ (Fig. 6). The relative intensities of the methine resonances of the estolide, the more downfield signal corresponding to the free C-18 alcohol, and the upfield free C-7 alcohol were approximately 1.9:0.6:2.6, indicating that the major site of branching is at the homoallyl C-18 alcohol, with an average of 1.9 estolide linkages per glycerol. For the triacylglycerol, two sets of doublet of doublet (dd) resonances for the methylene hydrogens of the glycerol backbone are located at δ 4.12 and 4.27 ppm (Fig. 6a). Benzoylation of Ov oil resulted in a second set of estolide methine signals (δ 5.1), mirroring observations with castor oil. Whereas in benzoylation at C-12 of castor oil resulted in typical two dd resonances for the methylene groups in the glycerol backbone (Fig. 6b), similar modification of C-7 resulted in new multiplet features and supported C-7 as the predominant location of unesterified OH groups (Fig. 6c).

**Figure 7:**
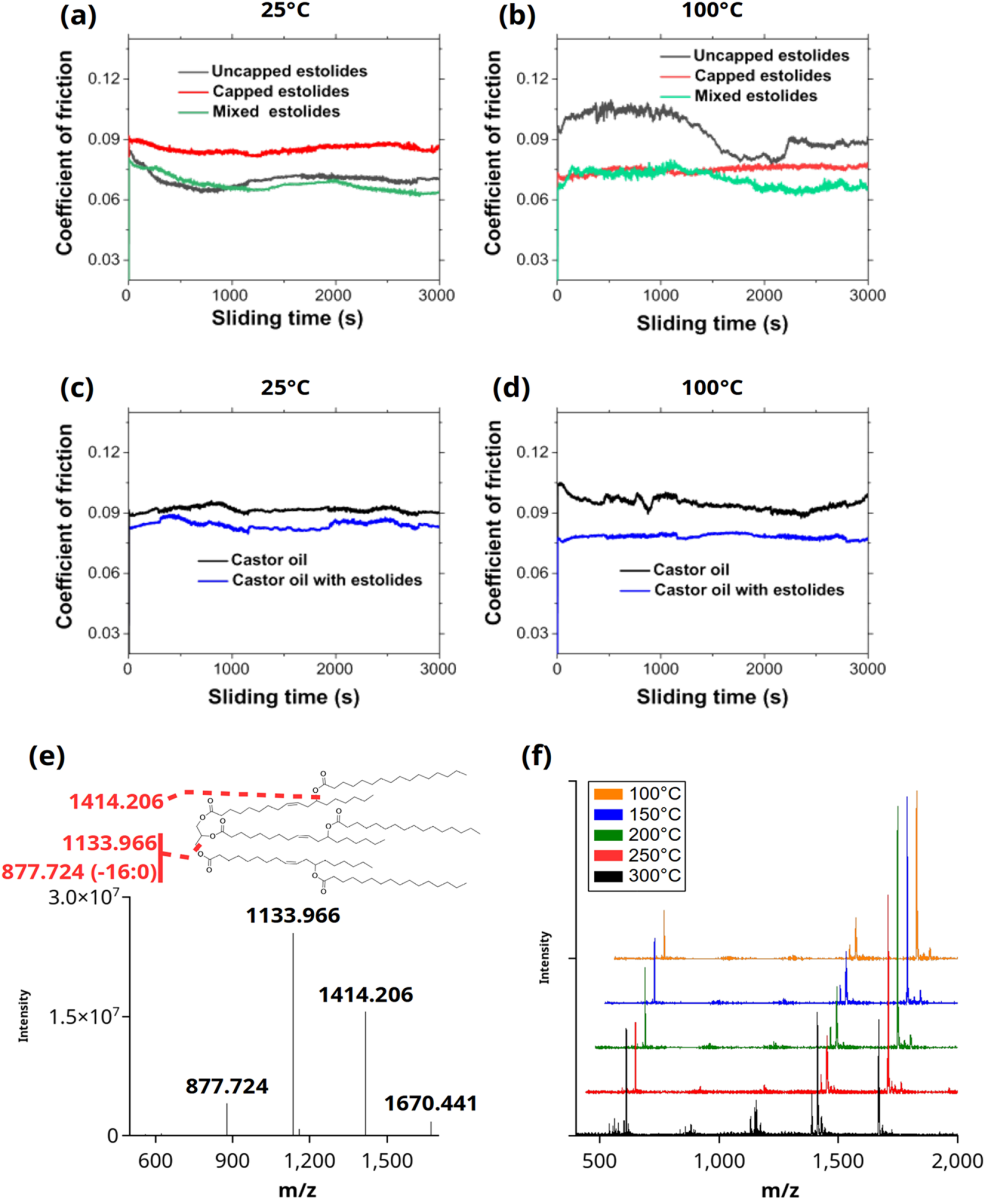
Lubrication properties of separated Ov estolides and synthetic castor estolides. Capped (red), uncapped (gray), and mixed (green) Ov TAG estolides showed different CoF at 25 °C (**a**) and 100 °C (**b**). Coefficient of friction results for castor oil with estolides (blue) and unmodified castor oil (black) at 25 °C (**c**) and 100 °C (**d**). (**e**) MALDI-MS/MS confirmed the structure of synthetic castor estolides with esterified 16:0. (**f**) ESI-MS of synthetic castor estolides showed fragmentation of the esterified 16:0 unlike the fragmentation of hydroxy TAG of unmodified castor oil, as seen in Fig. 4b.

TAG estolides terminating with an hFA are considered “uncapped” estolides while those terminating with a non-hydroxy FA are considered “capped” estolides. HPTLC separated the Ov TAG estolides by whether they were capped or uncapped and by the degree of acylation in the estolide branch chain (Fig. 5a, HPTLC bands 1–3 are capped, 4–6 are uncapped). Considering that uncapped TAG estolides may have different tribological properties in comparison to the properties of capped TAG estolides, the two types were chromatographically separated to determine CoFs at different temperatures (Fig. 7, Supplemental Fig. 6). At 25 °C the uncapped TAG estolides had a lower CoF relative to the capped TAG estolides, but at 100 °C capped TAG estolides showed a lower CoF (Fig. 7a,b). By contrast, at both temperatures a mixture of capped and uncapped TAG estolides (natural Ov oil) had a lower CoF, suggesting that both types of TAG estolides contributed to the improved tribological properties observed in Ov oil.

Previously, the improved properties of castor oil over other plant-derived oils were attributed to the presence of hFAs^33^. However, the unique estolide structures of Ov oil result in its superior performance over castor oil. With the knowledge that TAG estolides of Ov oil affect its performance, a path is suggested for improving lubrication characteristics of other plant-based oils. Here synthetic TAG estolides were made from castor oil by esterifying the hydroxyl groups of ricinoleate with palmitate (Fig. 7e), and then used to measure the CoF at 25 °C and 100 °C compared to unmodified castor oil. The synthetic castor estolides differed from Ov TAG estolides by being entirely “capped” with palmitate esterified to every available OH of tri-ricinolein. The synthetic castor estolides showed a lower CoF and wear at 25 °C and 100 °C relative to unmodified castor oil (Fig. 7c,d, Supplemental Fig. 7), consistent with previous results^21–23^, and further supporting the evidence that the presence of TAG estolides, and not only of hFAs, improve the tribological properties of plant seed oils. Even at higher temperatures, the synthetic castor estolides show better lubrication characteristics (Supplemental Fig. 8). The wear tracks of the steel surfaces used to measure CoFs of synthetic estoylated castor oil was also less than unmodified castor oil (Supplemental Figs 7, 9 and 10). Additionally, the synthetic castor estolides showed greater thermal stability compared to unmodified castor oil. At 300 °C fully estoylated tri-ricinolein showed fragmentation, decreasing its intensity, and subsequently increasing the intensity of synthetic castor estolides with 0, 1, or 2 estolide acylations (Fig. 7f).

The recent discovery of very long chain dihydroxy fatty acids in *O. violaceus* seeds prompted a preliminary functional examination of the seed oil^20^. It was originally presumed that the hFA content of the oil afforded the excellent high temperature lubricity properties. However, here we report that essentially all of the dihydroxy FA in *O. violaceus* oil is in the form of branched estolides esterified to TAGs, and we conclude that it is not hFA *per se*, but rather the estolide nature of the seed oil that is responsible for its enhanced lubricity properties, especially over castor oil. Discovering the estoylated nature of Ov oil served as a guide to further improve castor oil. This provides an example of designing a synthetic oil based on properties derived from a naturally occurring seed oil. Additionally, the observations of mixed, capped, and uncapped Ov TAG estolides suggest oil blends may produce favorable properties. Together this study revealed an unusual mixture of complex lipids in *Orychophragmus violaceus* seeds formed from dihydroxy VLCFAs to TAG estolides with a long polymerized estolide branch chain, and showed the chemistry of unusual lipids from nature provides new insights into designing and understanding synthetic oils for improved and varied properties. Finally, Ov seed oil may offer a source of a functional plant-based oil as an alternative to petroleum-based oils as well as a unique chemical feedstock for synthesizing other useful bioproducts, such as plant oil-based polymers.

## Methods

### Orychophragmus violaceus seed oil extraction

Approximately 30 mg of *O. violaceus* seeds were used per extraction for oil used in MS applications. Seeds were homogenized by bead beating for 45 sec with glass beads (Biospec Mini-Bead-Beater-16, Bartlesville, OK, USA) in 1 ml of 70 °C isopropanol with 0.01% butylated hydroxytoluene (BHT, w/v). An additional 1 ml of 70 °C isopropanol was added to wash out homogenization tubes and collected with homogenized sample. The homogenized seeds were incubated at 70 °C for 30 min to extract total lipids. Following incubation, 1 ml of CHCl_3_ and 0.45 ml of distilled water were added to each extraction. Samples were left to extract overnight at 4 °C. Extracted samples had an additional 2 ml of isopropanol, 1 ml of CHCl_3_, and 0.45 ml of water added before vortexing and centrifugation to sediment homogenized material. The supernatant was transferred to fresh tubes and then partitioned with the addition of 1 ml of CHCl_3_ and 2 ml of 1 M KCl. Partitioned samples were vortexed and centrifuged. The aqueous top layer was aspirated off, and this washing was repeated two more times. Following the wash, the organic layer was evaporated to dryness under nitrogen gas. Dried extracts were resuspended in 1 ml of CHCl_3_ until prepared for MS analysis.

### Tribology tests

The Ov oil used for tribology tests was extracted directly from the seeds of Ov plants by cold pressing and filtering through a paper filter (Sigma Aldrich). The density of the Ov oil was measured to be 0.905 g/cm^3^. The viscosity of the Ov oil measured with Brookfield DV-II + viscometer was 1209 centipoise. Density and viscosity of the cold-pressed castor oil used as a baseline for the comparison analysis were 0.959 g/cm^3^ and 612 centipoises, correspondingly. The dependence of the kinematic viscosity values for the oils on the temperature conditions is summarized in Supplemental Fig. 10. Pour point and volatility of the oils were measured according to the ASTM D97 and ASTM C681 standards correspondingly. A summary of the measured properties of the oils is presented in Supplemental Table 1.

The tribology tests were done using an Anton Paar macroscale pin-on-disk tribometer with the 10 μN sensitivity of the frictional force sensor. The tests were performed at least 3 times to ensure reproducibility of the results. The tribology tests were performed in a linear reciprocating mode with 10 mm running distance and 1 Hz frequency of reciprocating motion. For the elevated temperature tests performed in the range of 25 up to 300 °C, the temperature readings were demonstrating ± 1 °C accuracy and stability.

Testing of the lubrication efficiency of the Ov oil was performed using mirror-polished (roughness ~ 20–30 nm) 440 C stainless steel flat and ball (6 mm in diameter) samples. The samples were heat treated to demonstrate the maximum hardness of 58 ± 2 HRC. Both the substrate and counterbody were cleaned by acetone before running the tests. The samples were submerged with 1.5 ml of oil during the tribology tests. The tests were performed at a maximum contact pressure of 1.5 GPa, indicating the boundary lubrication regime.

### Characterization of the wear

After the tests, to perform further characterization of the wear tracks formed, the excess of oil was removed and the samples were rinsed with acetone followed by isopropanol.

The wear volume of wear scar on the pin side was calculated based on the following equations

1 $$V=(\frac{\pi h}{6})(\frac{3{d}^{2}}{4}+{h}^{2})$$

where *d* is the wear scar diameter, *r* is the radius of the ball, and

2 $$h=r-\sqrt{{r}^{2}-\frac{{d}^{2}}{4}}$$

The optical images of the wear tracks were acquired using a Zeiss optical microscope. The micrographs and Energy Dispersive Spectroscopy (EDS) mapping were done by using FEI Quanta 200 Scanning Electron Microscope (SEM) equipped with EDS. The oxidation of the wear tracks was further characterized by Raman analysis performed using Nicolet Almega XR Dispersive Raman spectrometer with a green laser (wavelength of 534 nm).

### HPTLC, ESI-MS, and MS/MS analysis of Ov oil

Extracts used for HPTLC separation were diluted 1:10 in CHCl_3_. Diluted lipid extract was spotted in a series of 2 μl spots on to an HPTLC plate (EMD Millipore HPTLC, Ca. no. 1.51160.0001). Spotted HPTLC plates were run in a solvent system of 70:30:1 diethyl ether/heptane/acetic acid. One lane of the HPTLC plate was cut off and then charred by spraying with an aqueous solution of 10% cuprous sulfate with 8% phosphoric acid. Sprayed cut section of the HPTLC plate was charred in a hot oven until dark bands were visible. The cut section of the HPTLC plate that was charred was used to guide the scraping of the bands from the uncharred portion of the HPTLC plate. Bands were scraped off the HPTLC plate using a razor blade. Scrapings for each apparent band were collected separately and then extracted with 1 ml of CHCl_3_/MeOH (1:1, v/v) three times. Extracted washes were collected together and evaporated under nitrogen until dryness until prepared for ESI-MS analysis. Full TLC image is included in the Supplementary Information (Supplemental Fig. 11).

Extracted seed oil and extracted lipids scratched off from HPTLC plate used in ESI-MS analysis were diluted and resuspended in 1:100 concentration in CHCl_3_/MeOH/500 mM ammonium acetate (1:1:0.02, v/v/v) prior to analysis. From seed oils assayed for thermal stability and friction coefficients, 30 mg of spent seed oil was massed and then dissolved 1:100 (wt/v) in CHCl_3_/MeOH (2:1, v/v) until prepared for ESI-MS analysis in which dissolved oil was diluted 1:100 further in CHCl_3_/MeOH/500 mM ammonium acetate (1:1:0.02, v/v/v) prior to analysis. Samples were analyzed by direct infusion ESI-MS using an API 3000 triple quadrupole mass spectrometer (Applied Biosystems). The following parameters were set during analysis: injection rate of 20 μl/min, source temperature of 100 °C, curtain gas of 10, nebulizing gas of 12, ionspray voltage of + 5500 V, declustering potential of 100 V, other parameters were left as default. Total ion scans were collected from m/z of 700 to 2500 with a scan time of 1.8 sec for extracted seed oils, and collected from m/z 200 to 700 and 700 to 2500 for spent seed oil used in thermal stability assays. Product ion scans were collected using the same set parameters with the following exceptions: collisional energy between 35 and 45 V, and collisional cell exit potential of 14 V. Samples used in product ion scans to determine OH binding of the estolide branch from the glycerol bound hydroxy FA in negative ionization mode were conducted with the same parameters described above with the following modifications: ionization mode set to negative, ion spray voltage of −4500 V, declustering potential of −60 V, collisional energy between −45 V and −60 V, collected from m/z 50 to 850 with a scan time of 1 sec. Data was collected using Analyst software (Sciex), exported as individual text files, and then analyzed.

### TAG estolide nomenclature

TAG estolides are referred to by a nomenclature with the following structure: aa:bb-nOH, where ‘aa’ refers to the number of C in the FA moieties, ‘bb’ refers to the number of unsaturations, and ‘n’ refers to the number of OH groups from the dihydroxy FAs present. For example, the uncapped diacyl TAG estolide of 108:8-6OH (Supplemental Fig. 5) contains 108 C in the FAs, 8 unsaturations, and 6 OH groups from the dihydroxy FAs. TAG estolides with hFAs at the terminal end of the estolide branch chain are called “uncapped”, while those TAG estolides with nonhydroxy FAs at the terminal end are “capped” TAG estolides.

### Solid phase extraction separation of Ov TAG estolides

To determine the individual tribological properties of capped and uncapped TAG estolides, each type was separated using solid phase extraction (SPE) on a Supelco Discovery DSC-Si 6 ml, SPE cartridge (Sigma Aldrich cat. no. 52655-U). Ov oil was extracted in the same manner described above from approximately 2 g of seeds. Oil extracts were dissolved in 2 ml of hexane and divided into four portions roughly representing 500 μl. Each divided portion was loaded on to an individual SPE cartridge and let flow through. The solvents used to elute the TAG estolides through the column include, in the order used: 6 ml of hexane/diethyl ether (4:1, v/v) collected in 0.5 ml fractions, 5 ml of methanol collected in 1 ml fractions, and 3 ml of chloroform collected in a single fraction. Following the collection of the fractions, 2 μl were spotted on to an HPTLC plate to estimate the efficacy of separation. Solvent conditions and detection for TLC analysis were the same as described above. Those fractions deemed to contain only capped or only uncapped TAG estolides were combined to form one collected sample of either capped TAG estolides or uncapped TAG estolides. Fractions containing a mixture of both capped and uncapped TAG estolides were pooled into a single collected sample to be used as a comparison to the samples containing only capped or uncapped TAG estolides. Additionally, another 1 g of Ov seeds were extracted and separated by SPE, but had all collected fractions combined as a control unseparated oil to compare to Ov oil separated into capped, uncapped, and mixed samples. In both the separated and unseparated oils, a waxy, resinous material eluted during the chloroform wash and was collected but not mixed into any of the collected or pooled fractions as its identity could not be determined by TLC. On the TLC plate, this fraction remained as a spot at the origin with no apparent migration. The separated TAG estolides were then used in tribological measurements to determine the properties of each kind of TAG estolides relative to the unseparated Ov oil, labeled as “mixed.”

### MALDI-MS and MS/MS analysis of Ov seed oil

Extracted *O. violaceus* oil was analyzed by MALDI-MS/MS using a MALDI-LTQ-Orbitrap-XL mass spectrometer (ThermoScientific) by spotting 5 μl of 1:10 oil diluted in CHCl_3_ on to a Superfrost Plus microscope slide (Fisherbrand), dried under a stream of nitrogen gas. Dried spots were coated with 2,5-dihydroxybenzoic acid (2,5-DHB) by sublimation. Mass spectrometer parameters were set as follows: laser energy of 12 μJ/pulse, 10 laser shots per step, normalized collision energy of 40, and an activation time of 35 ms. The MS/MS scan of the uncapped diacyl-estolide 108:8-6OH was selected for the sodiated parent ion of *m/z* 1778.50 and collected from a *m/z* range of 485 to 1800. The MS/MS scan of the uncapped triacyl-estolide 132:10-8OH was selected for the sodiated parent ion of *m/z* 2156.8 and collected from a *m/z* range of 590 to 2200. Data were averaged across 50 steps and exported from Xcalibur software (ThermoScientific).

### Synthesis of synthetic palmitoylated (16:0) estolides of castor oil

In a dry 250-mL one-necked round-bottom flask equipped with a septum and mineral oil bubbler, was placed a palmitic acid (40.44 g, 0.158 mmol, 3.62 equiv), toluene (40 mL), and a football-shaped stir bar. To the suspension, *N*,*N*-dimethylformamide (250 μL) was added to the suspension followed by oxalyl chloride (12.9 mL, 0.152 mol, 3.5 equiv) in five portions over one hour. During the course of the reaction, gas was rapidly evolved with very little heat production. After stirring for 4 h, a homogeneous, colorless solution resulted. Nitrogen gas was bubbled through the solution for 15 minutes to reduce the dissolved HCl content.

A second dry 250-mL one-necked round-bottom flask equipped with a dropping funnel and N_2_ gas inlet was loaded with castor oil (40.34 g, estimated molecular weight 933 g/mol, 0.0435 mol, 1.00 equiv), toluene (30 mL), and pyridine (12.7 mL, 0.158 mol, 3.62 equiv). The dropping funnel was loaded with palmitoyl chloride solution that was added over 25 min, using an ice-bath to moderate reaction temperature to < 30 °C. After stirring at 20 °C for 17 h, the resulting solution was extracted with toluene (20 mL) and water (75 mL). The organic phase was then washed with 50% (w/v) *N*,*N*-dimethylamino-2-ethanol (deanol; 3 × 25 mL) at 80 °C, water (2 × 50 mL), and 1 N HCl (100 mL then 50 mL). The organic phase and the upper portion of the aqueous phase were filtered through Celite to remove solids. The hazy solution was then washed with water (3 × 50 mL) and saturated brine (25 mL). After drying over MgSO_4_ and vacuum filtration, the solvent was removed at 50 °C using a rotary evaporator, the final yield of pale yellow estolide oil was 60.08 g (89%).

IR (KBr film, neat) 2922, 2852, 1734, 1465, 1166, 722 cm^−1^; ^1^H NMR (500 MHz, CDCl_3_) δ 5.4–5.5 (m, 2.71 H), 5.3 (m, 3.53 H), 5.24 (m, 1 H), 4.86 (m, 2.67 H, estolide RR′C**H**Opalmitoyl), 4.28 (dd, 2 H), 4.13 (dd, 2 H), 2.3 (m 19.6 H), 2.0 (m, 6.53 H), 1.6 (m, 12.4 H), 1.5 (m, 5.76 H), 1.2–1.35 (m, 123 H), 0.85 (m, 18 H); ^13^C{^1^H} NMR (125.7 MHz) δ 173.5, 173.2, 132.4, 124.4, 73.6, 68.9, 62.0, 34.7, 34.1, 34.0, 33.6, 32.0, 31.9, 31.7, 29.7, 29.62, 29.58, 29.52, 29.46, 29.32, 29.27, 29.15, 29.1, 29.05, 29.01, 27.3, 25.3, 25.1, 24.83, 24.80, 22.6, 22.5, 14.05, 14.00.

Benzoylation of castor and Ov oil was accomplished using a similar procedure.

## Supplementary information

Supporting Information

## Data Availability

The authors declare that data supporting the findings of this study are available within the article and its supplemental information files.

## References

[1] Holmberg K, Andersson P, Erdemir A (2012). Global energy consumption due to friction in passenger cars. Tribology International.

[2] Chu S, Majumdar A (2012). Opportunities and challenges for a sustainable energy future. nature.

[3] Berman D, Erdemir A, Sumant AV (2018). Approaches for Achieving Superlubricity in Two-Dimensional Materials. ACS Nano.

[4] Berman D, Deshmukh SA, Sankaranarayanan SKRS, Erdemir A, Sumant AV (2015). Macroscale superlubricity enabled by graphene nanoscroll formation. Science.

[5] Szeri, A. Z. *Tribology: Friction, Lubrication, and Wear* (Hemisphere 1980).

[6] Khonsari, M. M. & Booser, E. R. *Applied Tribology: Bearing Design and Lubrication*. (John Wiley & Sons, 2001).

[7] Brendow K (2003). Global oil shale issues and perspectives (Synthesis of the Symposium on Oil Shale held in Tallinn (Estonia) on 18 and 19 November 2002). Oil Shale.

[8] Keeble BR (1988). The Brundtland report:‘Our common future’. Medicine and War.

[9] Haus F, German J, Junter G-A (2001). Primary biodegradability of mineral base oils in relation to their chemical and physical characteristics. Chemosphere.

[10] Wilson B (1998). Lubricants and functional fluids from renewable sources. Industrial Lubrication and Tribology.

[11] Wu X, Zhang X, Yang S, Chen H, Wang D (2000). The study of epoxidized rapeseed oil used as a potential biodegradable lubricant. Journal of the American Oil Chemists’ Society.

[12] Bisht R, Sivasankaran G, Bhatia V (1993). Additive properties of jojoba oil for lubricating oil formulations. Wear.

[13] Bhatia V, Chaudhry A, Sivasankaran G, Bisht R, Kashyap M (1990). Modification of jojoba oil for lubricant formulations. Journal of the American Oil Chemists’ Society.

[14] Asadauskas S, Perez JH, Duda JL (1997). Lubrication properties of castor oil–potential basestock for biodegradable lubricants. Tribology & Lubrication Technology.

[15] Smith F (1959). Lubricant behaviour in concentrated contact systems—the castor oil-steel system. Wear.

[16] Sánchez R, Franco J, Delgado M, Valencia C, Gallegos C (2009). Development of new green lubricating grease formulations based on cellulosic derivatives and castor oil. Green chemistry.

[17] Liu S (2019). Renewable lubricants with tailored molecular architecture. Science Advances.

[18] Balakrishnan M (2015). Novel pathways for fuels and lubricants from biomass optimized using life-cycle greenhouse gas assessment. Proceedings of the National Academy of Sciences.

[19] Balakrishnan M (2016). Production of renewable lubricants via self-condensation of methyl ketones. Green Chemistry.

[20] Li X (2018). Discontinuous fatty acid elongation yields hydroxylated seed oil with improved function. Nature Plants.

[21] Cermak SC, Brandon KB, Isbell TA (2006). Synthesis and physical properties of estolides from lesquerella and castor fatty acid esters. Industrial Crops and Products.

[22] Hayes DG, Kleiman R (1995). Lipase-catalyzed synthesis and properties of estolides and their esters. Journal of the American Oil Chemists’ Society.

[23] Cermak SC, Isbell TA (2003). Synthesis and physical properties of estolide-based functional fluids. Industrial crops and products.

[24] Lin J-T, Arcinas A, Harden LR, Fagerquist CK (2006). Identification of (12-ricinoleoylricinoleoyl) diricinoleoylglycerol, an acylglycerol containing four acyl chains, in castor (Ricinus communis L.) oil by LC-ESI-MS. J. Agric. Food. Chem..

[25] Hayes DG, Kleiman R, Phillips BS (1995). The triglyceride composition, structure, and presence of estolides in the oils ofLesquerella and related species. Journal of the American Oil Chemists’ Society.

[26] Doehlert DC, Moreau RA, Welti R, Roth MR, McMullen MS (2010). Polar lipids from oat kernels. Cereal Chem..

[27] Plattner RD, Payne-Wahl K, Tjarks LW, Kleiman R (1979). Hydroxy acids and estolide triglycerides ofHeliophila amplexicaulis Lf Seed oil. Lipids.

[28] Smith MA, Zhang H, Forseille L, Purves RW (2013). Characterization of novel triacylglycerol estolides from the seed oil of Mallotus philippensis and Trewia nudiflora. Lipids.

[29] Mikolajczak K, Smith C (1968). Penta-acid triglycerides of Chamaepeuce afra seed oil. Biochimica et Biophysica Acta (BBA)-Lipids and Lipid Metabolism.

[30] Chang Q (2017). Operando formation of an ultra-low friction boundary film from synthetic magnesium silicon hydroxide additive. Tribology International.

[31] Erdemir Ali, Ramirez Giovanni, Eryilmaz Osman L., Narayanan Badri, Liao Yifeng, Kamath Ganesh, Sankaranarayanan Subramanian K. R. S. (2016). Carbon-based tribofilms from lubricating oils. Nature.

[32] Gosvami NN (2015). Mechanisms of antiwear tribofilm growth revealed *in situ* by single-asperity sliding contacts. Science.

[33] Bowden F, Gregory J, Tabor D (1945). Lubrication of metal surfaces by fatty acids. Nature.

