# Supplementary material for: Nature-Guided Synthesis of Advanced Bio-Lubricants
Source: Sci Rep. 2019 Aug 12;9:11711. doi: 10.1038/s41598-019-48165-6 (PMC6690888; doi:10.1038/s41598-019-48165-6)
Supplement: Supplementary file 1 — Supporting Information [file 41598_2019_48165_MOESM1_ESM.pdf]

Supplemental Information

Nature-Guided Synthesis of Advanced Bio-Lubricants

Trevor Romsdahl, Asghar Shirani, Robert E. Minto, Chunyu Zhang, Edgar B. Cahoon, Kent Chapman, Diana Berman.

[1] BioDiscovery Institute and Department of Biological Sciences, University of North Texas, Denton, TX, USA

[2] Department of Materials Science and Engineering, University of North Texas, Denton, TX, USA

[3]Department of Chemistry and Chemical Biology, Indiana University-Purdue University Indianapolis, Indianapolis, IN, USA.

[4] National Key Lab of Crop Genetic Improvement and College of Plant Science and Technology, Huazhong Agricultural University, Wuhan, China

[5]Center for Plant Science Innovation & Department of Biochemistry, University of Nebraska-Lincoln, Lincoln, NE, USA

*Correspondence and request for materials should be addressed to: Kent D. Chapman at kent.chapman@unt.edu or Diana Berman at diana.berman@unt.edu.

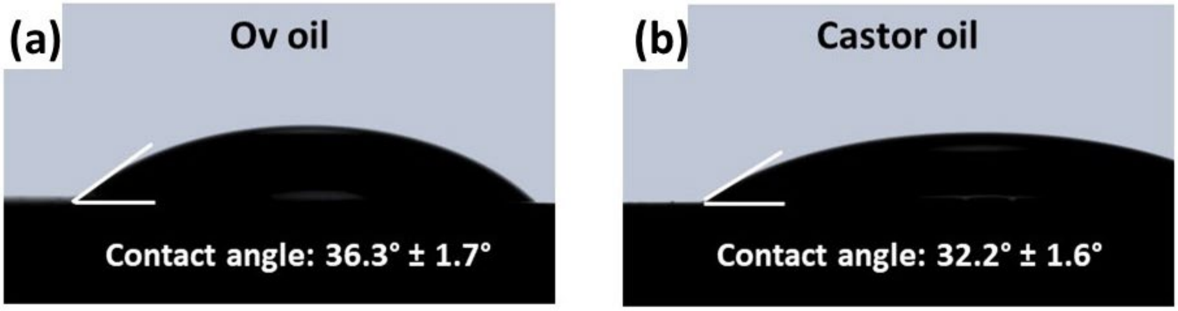

Supplemental Figure 1. Wetting characteristics of Ov oil in comparison to castor oil. Both (a) Ov oil and (b) castor oil create low contact angle with underlying steel substrate, thus further supporting high lubricity potential.

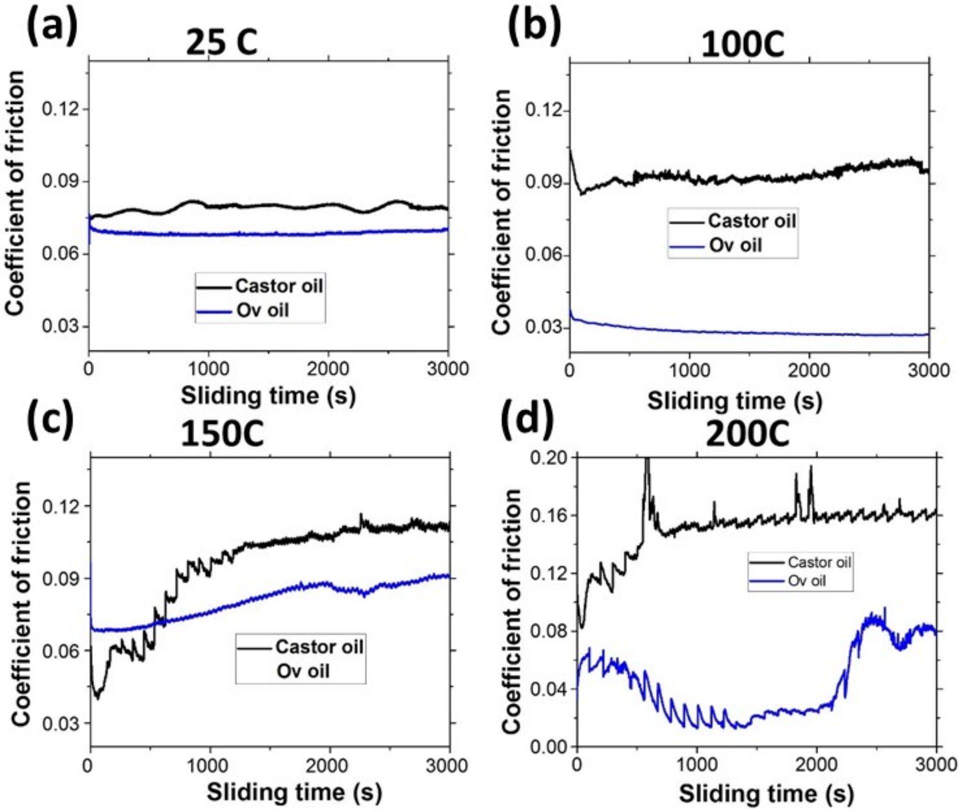

Supplemental Figure 2. Tribological analysis of the Ov oil in comparison to castor oil. Tribology behavior of the Ov and castor oils at (a) 25 °C, (b) 100 °C, (c) 150 °C and (d) 200 °C.

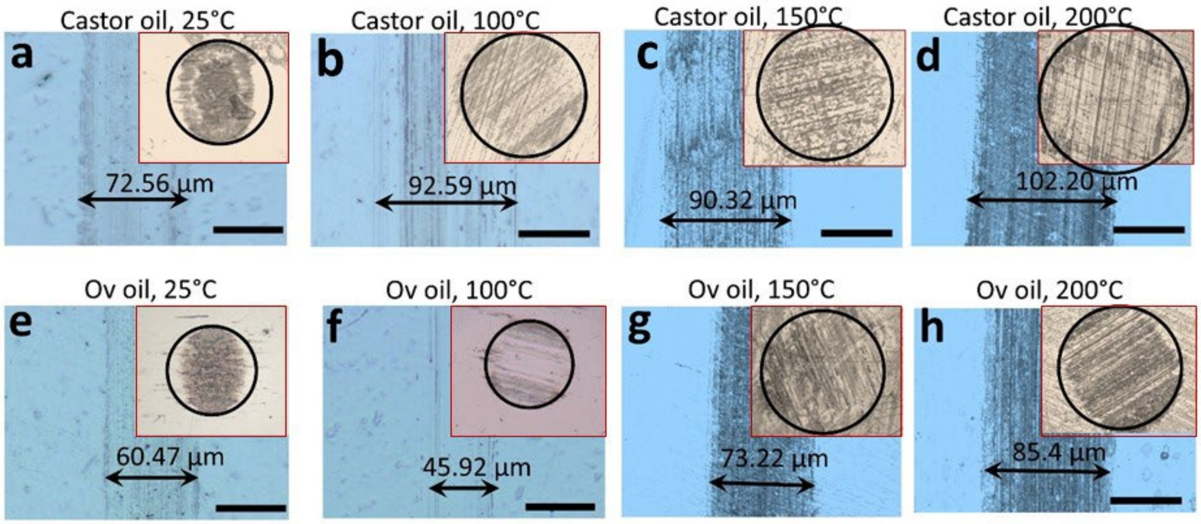

Supplemental Figure 3. Wear tracks and wear marks formed on steel surfaces during tests in Ov and castor oils. Analysis of the wear tracks for the tests performed in castor and OV oils, correspondingly, at (a) and (e) 25 °C, (b) and (f) 100 °C, (c) and (g) 150 °C, and (d) and (h) 200 °C.

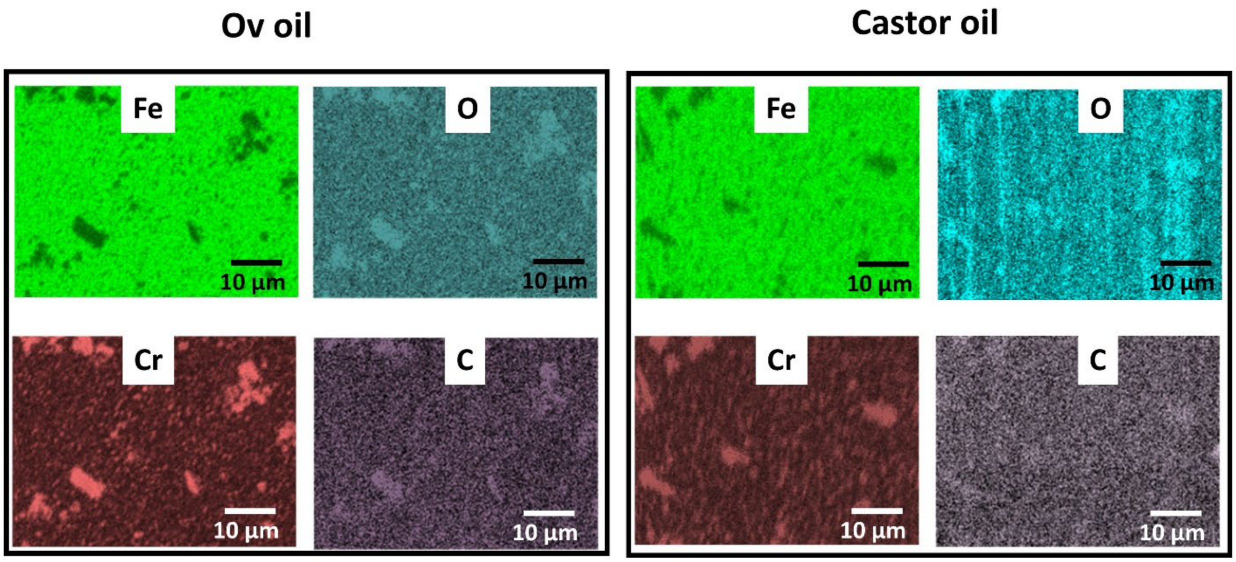

Element	At %
Fe	63.16
Cr	15.07
C	19.12
Si	0.30
Mo	0.22
O	2.13

Element	At %
Fe	55.86
Cr	13.65
C	24.10
Si	0.45
Mo	0.22
O	5.72

Supplemental Figure 4. SEM-EDS elemental maps of Ov and castor oil wear tracks for chromium and iron. The wear track in case of the castor oil indicates larger concentration of oxygen and carbon content association (scale bar = 10 µm).

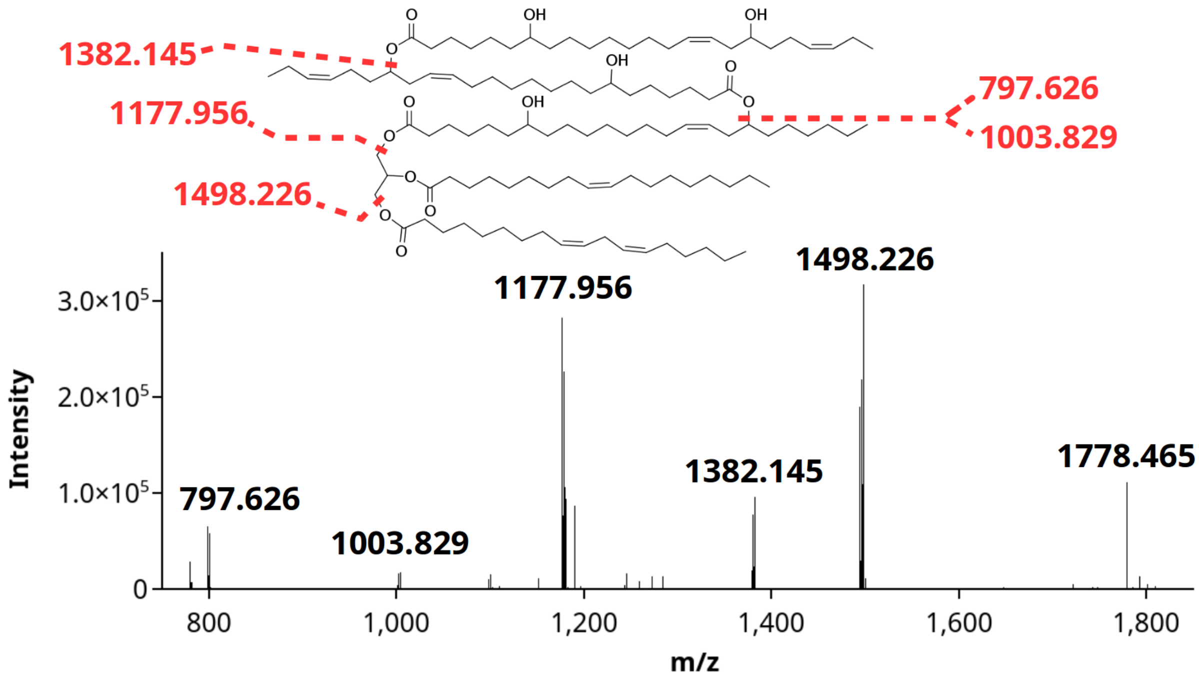

Supplemental Figure 5. MALDI-MS/MS of uncapped diacyl TAG estolide 108:8-6OH. The parent ion mass of 1778.5 corresponded to the sodiated parent ion mass of an uncapped diacyl TAG estolide with the composition of 108:8-6OH. Strong fragment ion signals were found at *m/z* 1498.226, 1382.145, 1175.939, 1003.829, and 797.626. The fragment ion at 1498.226 indicated a loss of a linoleate ion $[M - 18{:}2 + Na]^+$. Additional peaks differing by approximately 2 amu were also found at *m/z* 1496.212 and 1494.195 suggesting fragmentation of oleate $[M - 18{:}1 + Na]^+$ and stearate $[M - 18{:}0 + Na]^+$, respectively, from overlapping isobaric species. Isobaric TAG estolide molecular species are also overlapping in the ESI-MS spectrum scan as seen in Figure 3A. Fragmentation at 1382.145 and 1380.129 indicated a loss of a wuhanic acid $[M - 24{:}2(OH)_2 + Na]^+$ or nebraskanic acid $[M - 24{:}1(OH)_2 + Na]^+$, respectively. The fragment of the estolide branch was seen at *m/z* 1175.939 consisting of three wuhanic acids from the glycerol backbone. This suggested the estolide branch chain is likely from a single glycerol bound hFA rather than multiple glycerol bound hFAs. A TAG fragment ion was seen at *m/z* 1003.829 resulting from the fragment loss of two wuhanic acids $[M - 2(24{:}2(OH)_2) + Na]^+$. An *m/z* peak at *m/z* 1001.814 indicated a loss of one wuhanic acid and one nebraskanic acid $[M - 24{:}2(OH)_2 - 24{:}1(OH)_2 + Na]^+$, which is due to overlapping isobaric TAG estolides in the MS/MS spectrum. Fragment ions at *m/z* 797.626 and 799.641 suggested fragment ions of an estolide branch from the glycerol backbone consisting of two wuhanic acids $[(24{:}2(OH)_2)_2 + Na]^+$ or of one wuhanic acid and one nebraskanic acid $[(24{:}2(OH)_2 + 24{:}1(OH)_2) + Na]^+$. The presence of fragmentation peaks at both *m/z* of 797.626 and 1175.939 support a TAG estolide structure with a single glycerol bound hFA with an estolide branch chain rather than multiple hFAs bound to the glycerol backbone.

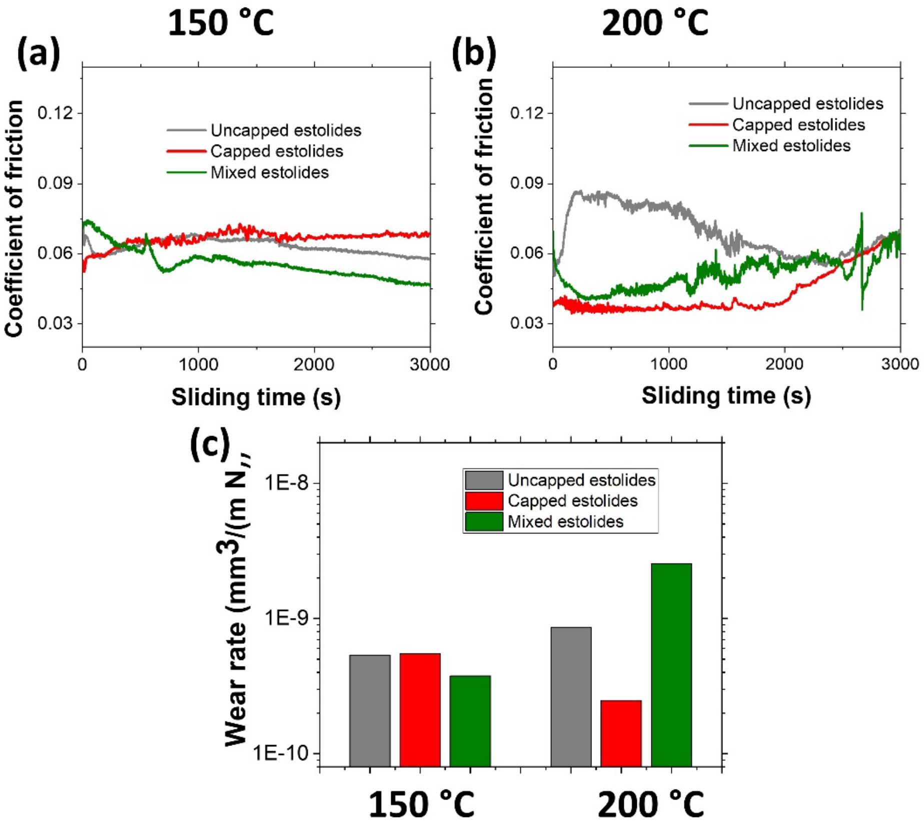

Supplemental Figure 6. Lubrication characteristics of Ov oil estolides at elevated temperature. Coefficient of friction at (a) 150 °C and (b) 200 °C and (c) corresponding wear rate measurements for separated from the Ov oil uncapped, capped, and mixed together uncapped and capped estolides.

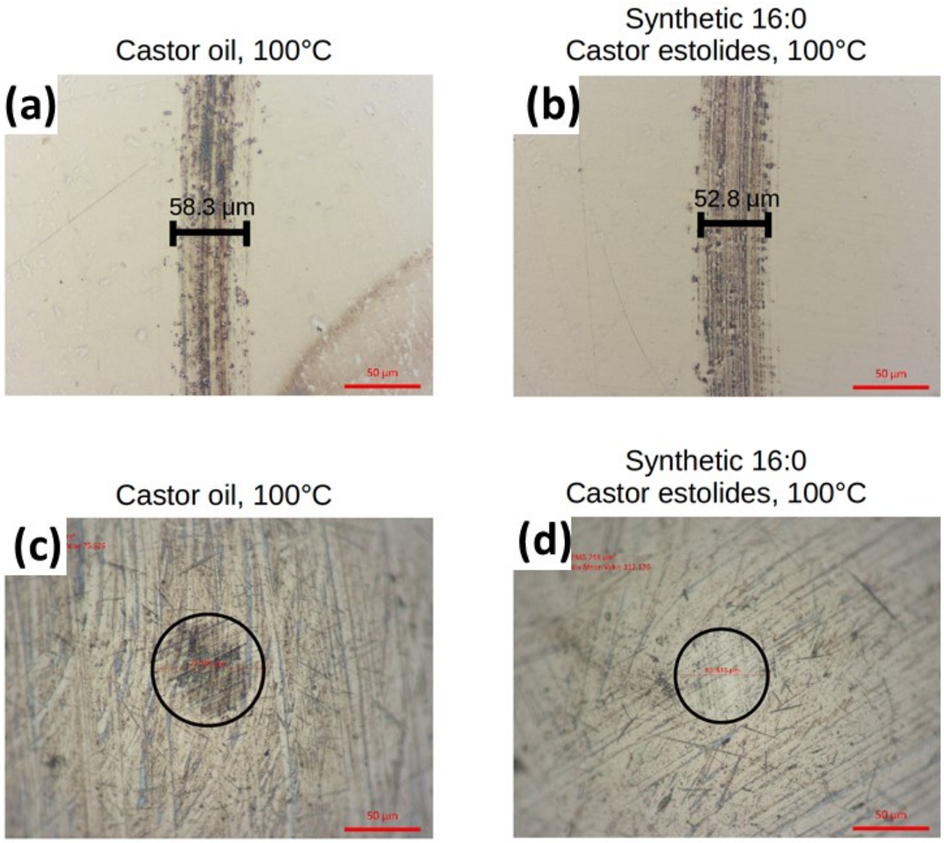

Supplemental Figure 7. Wear marks of purified Castor oil and synthetic 16:0 castor estolides. The synthetic castor estolides produced less wear during tribotests for both surface tracks (a & b) and ball tracks (c & d) (scale bar = 50 µm). The surface track width for unmodified castor oil (a) measured 58.3 µm versus 52.8 µm for synthetic castor estolides (b), and the ball track diameters measured 72.2 µm for unmodified castor oil (c) compared to (d) 61.6 µm for the synthetic castor estolides.

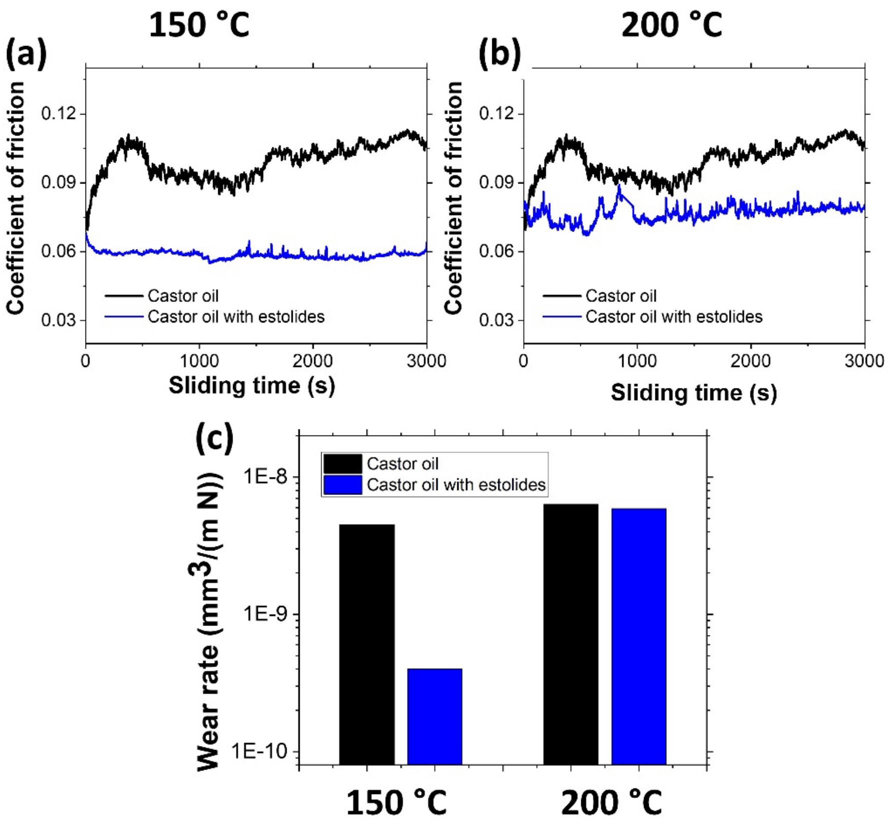

Supplemental Figure 8. Lubrication characteristics of purified castor oil and synthetic 16:0 castor estolides at elevated temperature. Coefficient of friction at (a) 150 °C and (b) 200 °C and (c) corresponding wear rate measurements.

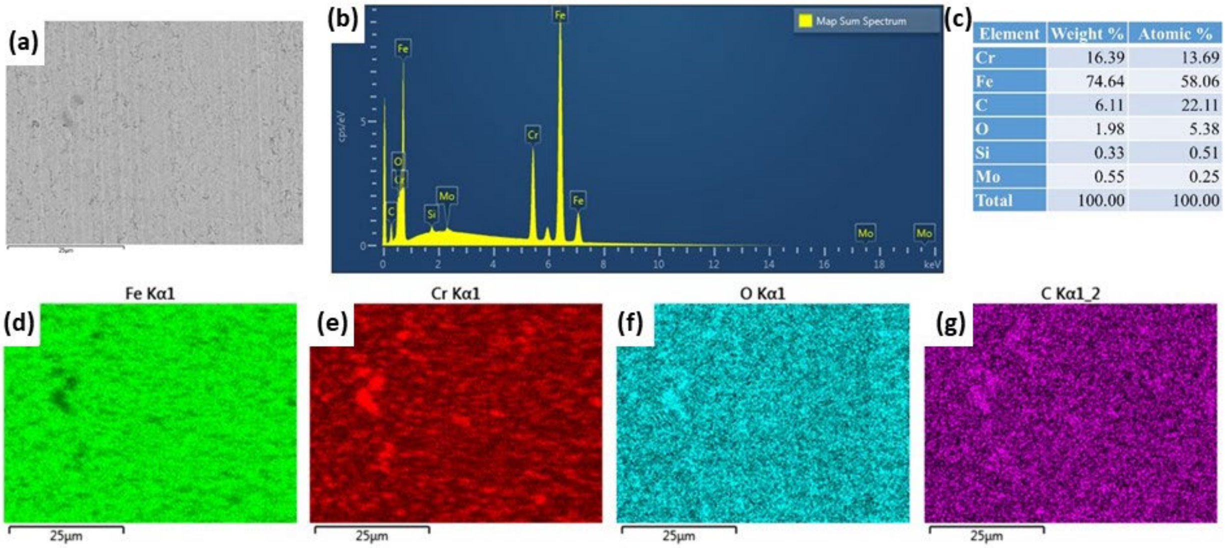

Element	Weight %	Atomic %
Cr	16.39	13.69
Fe	74.64	58.06
C	6.11	22.11
O	1.98	5.38
Si	0.33	0.51
Mo	0.55	0.25
Total	100.00	100.00

Supplemental Figure 9. Analysis of the wear track formed in synthetic 16:0 castor estolides. SEM EDS analysis of the (a) wear track formed during the tribological test: (b) detailed spectra acquired from the wear track with (c) relative concentration of different elements. Detailed map of (d) iron, (e) chromium, (f) oxygen, and (g) carbon atoms shows almost no contrast, thus indicating efficient lubricative nature of the estolides.

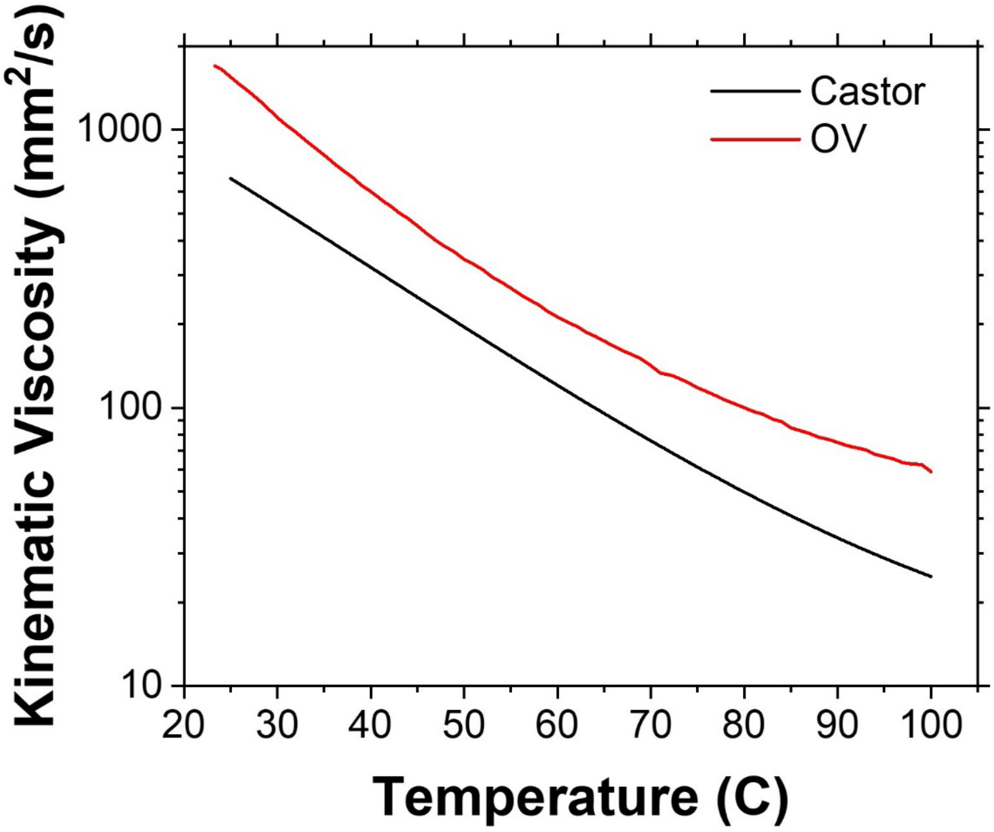

Supplemental Figure 10. Kinematic Viscosity measurements of castor and Ov oils. The results demonstrate difference in viscosity changes as a function of temperature.

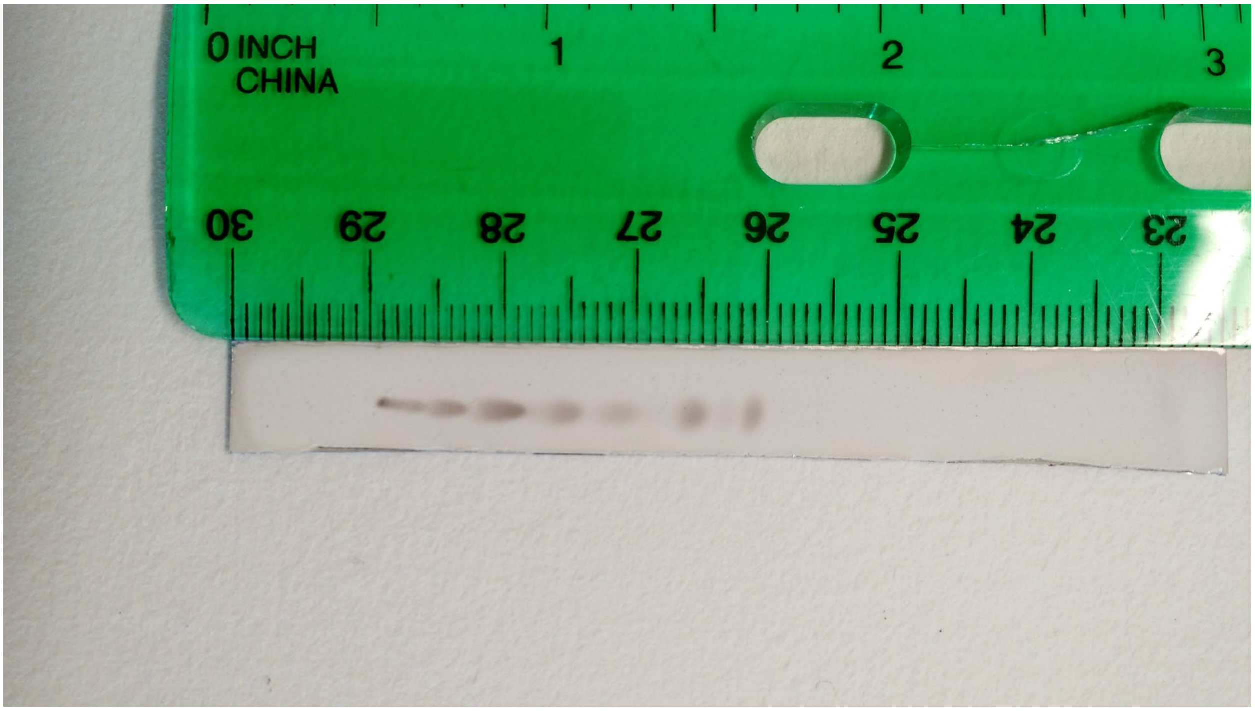

Supplemental Figure 11. Non-cropped TLC image.

Supplemental Table 1 Density, viscosity, and volatility characteristics of castor and Ov oil.

	**Castor Oil**	**Ov oil**
Density (g/mL)	0.959	0.905
Viscosity (mPa s) at RT	612	1209
Viscosity index	104.2	174.3
Pour Point (°C)	-21	-12
Volatility (%)	1.025	0.77
